# Age-Related Frontal Hyperactivation Observed across Different Working Memory Tasks: An fMRI Study

**DOI:** 10.3233/BEN-2012-120280

**Published:** 2012-08-09

**Authors:** Mohammad Fakhri, Hajir Sikaroodi, Farid Maleki, Mohammad Ali Oghabian, Hosein Ghanaati

**Affiliations:** ^1^NeuroImaging and Analysis GroupResearch Center for Molecular and Cellular ImagingTehran University of Medical SciencesTehranIran; ^2^School of MedicineShahid Beheshti University of Medical SciencesTehranIran; ^3^Department of NeurologyShariati HospitalTehran University of Medical SciencesTehranIran; ^4^NeuroImaging and Analysis GroupResearch Center for Molecular and Cellular ImagingTehran University of Medical SciencesTehranIran; ^5^Medical Imaging CenterImam Khomeini HospitalTehran University of Medical SciencesTehranIran

**Keywords:** Working memory, fMRI, task, stimulus type, age

## Abstract

*Purpose:* To evaluate patterns of activation, convergence and divergence of three functional magnetic resonance imaging (fMRI) Working Memory (WM) tasks in two different age groups. We want to understand potential impact of task and subjects’ age on WM activations as well as most important areas with regard to WM functions.

*Materials and methods:* Thirty-five healthy volunteers completed visual, verbal, and novel auditory WM tasks. The subjects were selected from age extremes to depict possible impact of normal aging. The General Linear Model was used to report significant activations and the effect of age group. Contrasts revealed differences in activation between tasks, and Combined Task Analysis was performed to determine common regions of activation across tasks.

*Results:* Most of the observed differences between the tasks were seen in areas that were responsible for feature processing. Frontal regions were mainstay activation areas, regardless of the utilized stimulus. We found an age-related reduction in activity of visual (in visually-presented tasks) and auditory (in auditory task) cortices but an age-related increase in prefrontal cortex for all tasks.

*Conclusion:* Regardless of the type of the task stimuli, frontal regions are the most important activation areas in WM processing. These areas are also main targets of age-related changes with regard to activation patterns. Our results also indicate that prefrontal overactivity in working memory might be a compensatory effort to mask age-related decline in sensory processing.

## 1. Introduction

Working memory, as the ability to maintain and manipulate information in short intervals, is widely used in understanding cortical functions in the healthy brain. This memory also serves as an important item to study in neurologic diseases such as dementia [1–3]. Variability of the results across studies on normal populations is one of the most confining factors that limit use of these functional maps to be studies compared to those of the patients.

In the literature, different stimuli have been applied to demonstrate working memory organization in both healthy and diseased brains. The use of different stimuli and distinct tasks to activate the working memory network may yield to different functional maps [4, 5]. Alternatively, subjects' characteristics might exert a far-reaching influence on the activated areas. Age

ISSN 0953-4180/12/$27.50 © 2012 – IOS Press and the authors. All rights reserved is one of the most studied characteristics to delineate a possible impact on working memory processes [6]. A downward trajectory across the adult life span in non-demented individuals in working memory has been previously reported [7]; however, a long-standing controversy in the working memory aging literature roots questions whether the effects of aging on various cognitive functions have the same common cause or several different causes. Age-related decreases in occipital cortex activity have been attributed to inefficient sensory processing in the ventral (occipito-temporal) pathway. Therefore, most of the studied tasks in these reports use a visually-presented stimulus [8]. These findings are usually attributed to sensory (visual) decline in normal aging process. Moreover, several studies have found an age-related increase in prefrontal cortex activity across a variety of visually-presented working memory tasks. It has been suggested that during perception, older adults might compensate for deficits in sensory processes mediated by occipital regions by recruiting strategic processes mediated by prefrontal regions. To date, there is no report, which studies age-related activations in auditory working memory. Functional mapping of these physiologic age-related changes are imperative to make meaningful comparisons between healthy and patient populations. Understanding segregation and convergence of activated brain regions in different task presentations, together with age's impact on working memory, are mandatory steps in this process.

The goal of the present fMRI study is to highlight any convergence or difference between various stimulus types in triggering fMRI network. Our study uses three different tasks to activate working memory, including well-studied visual and verbal stimuli, and an auditory task. We were thus interested in exploring age-related differences in activity that occurred in each working memory task. On the basis of prior findings, we hypothesize that all three tasks would show age-related decreases in sensory activity (visual cortex or auditory cortex depending on the stimulus) and age-related increases in frontal region activity as a compensatory change.

## 2. Materials and methods

### 2.1. Subjects

Participants were 35 healthy volunteers in two age categories. The young group consisted of 19 (10 men, 9 women) right-handed subjects between the ages of 16 and 29 years (mean = 21 years, SD = 3.7 years). The older age group consisted of 16 healthy right-handed volunteers aged 55 to 78 years (mean = 68 years, SD = 7.9 years). A neurologist examined subjects in the second age group to ensure none of the participants had a positive history of previous neurologic or psychiatric complaints and examinations were negative for significant neurologic or hearing problems. Mini Mental State Examination (MMSE) scores were within the normal range for all subjects. All participants indicated strong right-hand preferences (mean laterality quotient = +71 [+48 to +82]) on the Edinburgh Handedness Inventory. Participants gave written informed consent and all of the procedure was explained clearly. The Scientific and Medical Ethics Committee of Tehran University of Medical Sciences approved the entire study protocol. All of the subjects were trained on the task to ensure comprehension and provide them with practice prior to the imaging scans.

### 2.2. Experimental design

The participants were supine on the scanner table, able to see the stimuli via a magnetic resonance (MR)-compatible liquid crystal display (LCD) on a pair of glasses. The stimuli were projected with the Presentation TM software version 0.6 (Neurobehavioral Systems; Palo Alto, CA, USA). Three different tasks were utilized to activate working memory network. Each subject was scanned on all three tasks and the order of task performance was counterbalanced (Fig. 1):

### 2.3. Visual working memory

For the visual task, a simple dot in the middle of the screen was presented for 100 milliseconds (ms) as a fixation period. Then subjects viewed non-colored and meaningless random polygons from a pool of pictures for 200 ms. All of the presented pictures consisted of a three-polygon item. After a retention interval of 900 ms, when the fixation dot appeared again, a test display containing one probe item was presented. Subjects were asked to determine, within 1300 ms, whether or not the probe item was the same as the sample item. This timing was formerly used to activate visual working memory [9]. The activation (A) block consisted of 10 rounds of this 2.5 seconds trial. The 25 second activation block was followed by a 25 seconds rest (R) block in which fixation dot was presented. Total scan time was 200 seconds in sequences of RARARARA. This array was applied for all three tasks (Fig. 1-A).

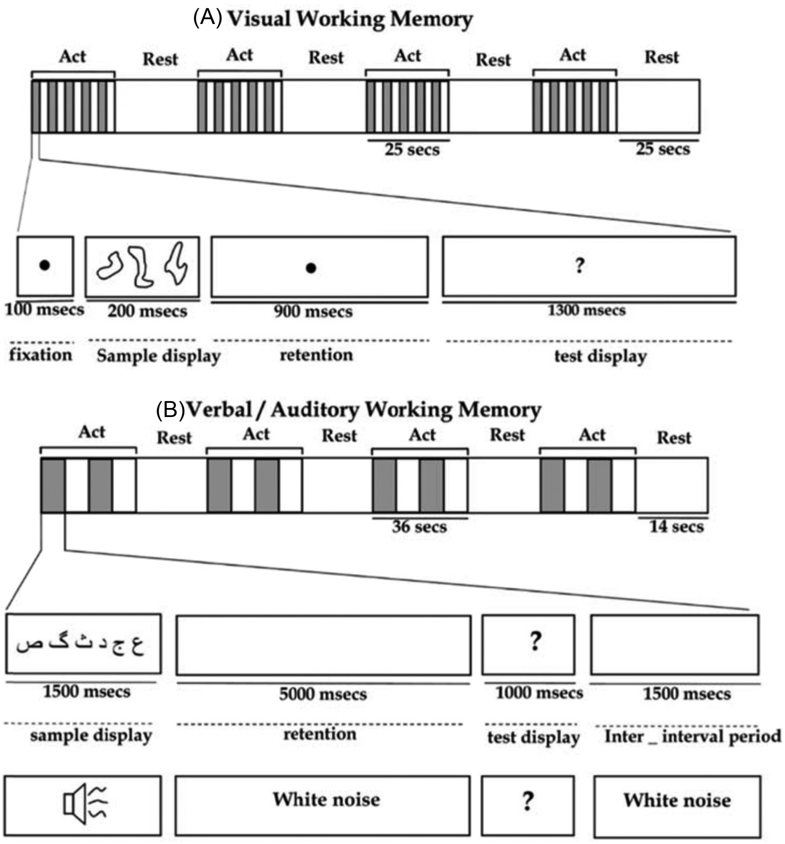

Fig. 1. Illustration of task design. A: visual working memory. B: verbal and auditory working memory tasks. Question marks mean "decision time" for the subjects.

### 2.4. *Verbal working memory*

To test verbal working memory, a typical Sternberg task was utilized. Subjects were presented with a 6-letter set of Persian alphabet for 1500 ms. Persian was the first language of all participants. All of the letters were consonants and distinct. A retention interval of 5000 ms with a blank screen was the next step. Then, a single probe letter would be appearing for 1000 ms. Participants were asked to decide whether or not the probe letter was one of the stimuli in the memory set. A 1500 ms blank screen indicated the end of this trial. Four rounds of this 9 second trial (36 seconds in total) would form the activation block for the verbal task. A 14 seconds rest block preceded every activation block. The scan would last for 200 seconds (RARARARA). A simple illustration of this task is presented in Fig. 1-B.

### 2.5. *Auditory working memory*

Auditory stimuli were presented with a computer and a pneumatic audio system. Participants kept their eyes closed during all conditions. As the scanner noise might exert a significant influence on the fMRI results, this study came up with a novel solution to mask the noise: we used white noise with a flat power spectral density to mask the baseline noise of the scanner. For the resting block and inter-trial intervals, this white noise was played. Stimuli in auditory working memory tasks were digitally synthesized 1000 to 3000 Hz tones. These tones had a two-part pattern, with a 150 ms gap embedded in different places of a 1500 ms interval. Hence, each auditory task unit lasts 1500 ms. A pool of this 1500 ms sound were previously generated and randomly used for stimulus presentation. The two components shared a similar frequency and the frequency remains constant across trials. Stimuli were delivered to the listener at 80 dB sound pressure level using MR-compatible headphones. For the activation and rest blocks, timing similar to that of verbal memory task was utilized. As stated earlier, in this task, white noise was used instead of blank screen (Fig. 1-B). To investigate the long-term reproducibility of fMRI activation

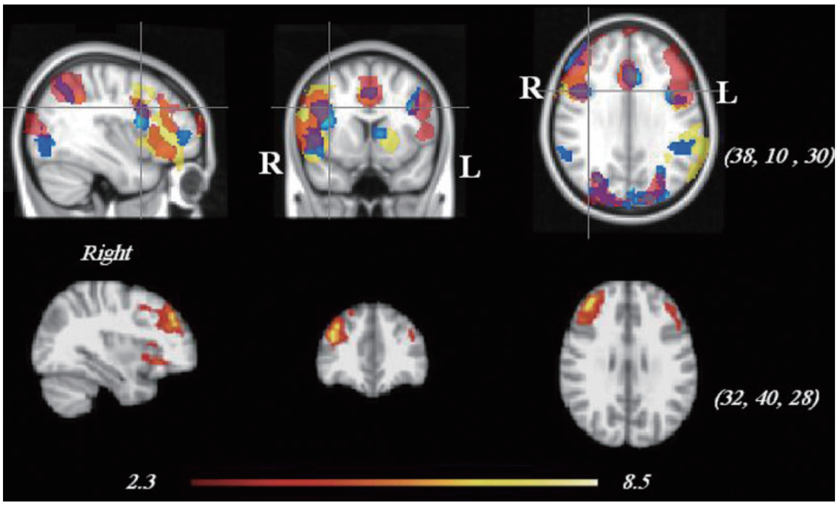

Fig. 2. Top: Group activation map for the visual, verbal and auditory working memory in all subjects (both groups). Bottom: Combined task analysis that shows frontal regions as shared activation across the tasks. Numbers in parentheses indicate "x", "y" and "z" levels on sagittal, coronal, and axial sections.

of this novel auditory working memory task, before the main imaging session, ten normal subjects performed the task. The experiment was repeated five times with the same settings for image acquisition and the fMRI task. Simple multi-subject analyses with a fixed-effect model were performed to make grouped session-specific functional maps for the ten subjects who were followed. Visual observation of the multi-subject activation maps showed similar activation patterns, and quantitative analysis showed small coefficients of variance of activation within regions of interest over time.

During all task presentations (200 seconds), 64 time series images were acquired (TR = 3125 ms).

An fMRI-compatible response pad was placed under the right hand of the participants and they were instructed to press a button using their right index finger only when the test probe was identical to the stimuli presented before the retention interval.

### 2.6. *Functional MRI and MRI data acquisition*

All participants were screened for the following exclusion criteria for fMRI experimentation: (I) ocular problems affecting the visual acuity at the time of scan; (II); ochlear implants or any metal objects in the body; (III) cardiac or neural pacemakers; and (IV) a history of musculoskeletal disorder in any limb. The MRI apparatus was a 1.5-Tesla GE® Signa scanner (General Electric; Milwaukee, WI, USA). A T1-weighted spin-echo sequence was used to generate high-resolution structural maps of participants' brains with the same dimension and orientation as the fMRI (TR = 1800 ms; TE = 90 ms; flip angle = 90°). The fMRI data were obtained with a gradient-echo echoplanar images (EPIs) protocol (TE = 60.3 ms; TR = 3125 ms; flip angle = 90°; field of view = 22 $cm^2$; number of slices = 15; slice thickness = 6 mm; spacing = 0 mm; bandwidth = 5.62 kHz). A standard quadrate head coil was used. Fifteen contiguous axial slices, relatively parallel to the "anterior commissure-posterior commissure" line according to the Talairach and Touroux atlas [10], were taken beginning from the vertex. The field of view was positioned to maximize coverage of the frontal and temporal lobes. Cerebellum was not included in the field of view.

### 2.7. *Task performance and functional analyses*

Performance of each subject was assessed by responses obtained during the scanning. In all tasks, one-third of the "test displays" were identical to "sample displays" and these matched probes were randomly inserted across the whole trial. Both wrong answers and missed answers were treated equally as errors. Repeated measures ANOVA assessed the effect of age, three tasks-types and age × task-type interaction on behavioral performance. Dependent measures were accuracy and median reaction time.

This study used FMRI Expert Analysis Tool (FEAT), part of FMRIB's Software Library (FSL, http://www.fmrib.ox.ac.uk/fsl), to perform statistical analyses. The following pre-processing steps were performed on EPI

Table 1
Significant activations in each task

| | Region[1] | Max Z stat | MNI[2] coordinate (x, y, z) |
|---|---|---|---|
| Verbal WM | | | |
| | Cingulate gyrus, Ant. division | 4.82 | (4, 18, 38) |
| | Lt Middle frontal gyrus | 4.4 | (−38, 2, 30) |
| | Rt Inferior frontal gyrus | 4.51 | (44, 8, 30) |
| | Lt Inferior frontal gyrus | 3.98 | (−46, 9, 31) |
| | Rt Frontal pole | 4.66 | (40, 48, 2) |
| | Lt Supramarginal gyrus | 3.32 | (−56, −30, 34) |
| | Lt Thalamus | 4.38 | (−12, 0, 8) |
| | Lt Precuneous cortex | 4.34 | (−12, −82, 44) |
| | Intracalcarine cortex | 6.33 | (6, −82, 2) |
| | Lt Lingual gyrus | 5.01 | (−24, −70, −2) |
| | Rt Lingual gyrus | 6.44 | (18, −82, −2) |
| Visual WM | | | |
| | Cingulate gyrus, Ant. division | 5.94 | (6, 6, 44) |
| | Rt Superior frontal gyrus | 4.82 | (6, 48, 44) |
| | Rt Middle frontal gyrus | 6.0 | (40, 28, 44) |
| | Lt Precentral gyrus | 4.86 | (−52, −4, 44) |
| | Occipital pole | 7.21 | (6, −98, 6) |
| | Lt Angular gyrus | 6.16 | (−40, −58, 42) |
| | Rt Lateral occipital cortex | 6.14 | (34, −62, 40) |
| Auditory WM | | | |
| | Rt Superior temporal gyrus | 6.74 | (58, −8, −2) |
| | Lt Superior temporal gyrus | 6.67 | (−52, −40, 18) |
| | Rt Inferior frontal gyrus | 6.62 | (48, 36, 16) |
| | Cingulate gyrus, Ant. division | 4.42 | (2, 26, 32) |
| | Lt Precentral gyrus | 5.69 | (−44, −6, 36) |
| | Rt Middle frontal gyrus | 4.65 | (46, 18, 36) |
| | Lt Parietal operculum cortex | 4.48 | (−56, −38, 26) |

[1]Regions with statistically significant activations are labeled according to Harvard–Oxford Cortical and Subcortical Structural Atlases.
[2]X, Y and Z demonstrate Montreal Neurological Institute (MNI) coordinates of maximum Z value within each region. WM = working memory; Ant = Anterior; Rt = Right; Lt = Left.

data before final analyses: motion correction, using Motion Correction from FMRIB's Linear Image Registration Tool (MCFLIRT; FSL), removal of non-brain tissue signals from anatomical images, using the Brain Extraction Tool (BET, Version 2.1; FSL), and spatial smoothing, using a Gaussian kernel of full-width half-maximum 8 mm and non-linear high-pass temporal filtering. The parametric statistical analysis was based on a general linear model, using FEAT, version 5.90; FSL [11].

Time-series statistical analysis was carried out using FILM pre-whitening (FMRIB Improved Linear Model) to make the statistical approaches valid and maximally efficient. Each voxel was analyzed against the convolved model with a resultant parameter estimate image. The parameter estimate is proportional to the signal change arising from the stimulus. Cluster thresholding with significance estimation defined by Gaussian Random Field Theory was used to identify clusters of activated voxels ($z$-score $> 2.3$; $p < 0.01$). To perform multi-subject (higher level) analyses, we registered the functional images to the standard space defined by the MNI-152 atlas [10]. Using FLIRT [12], we registered T1 images to standard stereotactic space, using the Montreal Neurological Institute (MNI) templates implemented in FSL and then we made EPI-to-T1 and T1-to-standard registrations. The contrast images obtained in first level analysis were then subjected to random effects analyses for the whole group, with age as a covariate [13]. Higher-level analysis for inter-subject (intra-paradigm) study was carried out using FLAME (FMRIB's Local Analysis of Mixed Effects) [14] stage 1 only (i.e., without the final MCMC-based stage). For an exploration of differences between the tasks, we used a paired t-test to identify areas activated more strongly in one task than the other.

We also used Combined Task Analysis (CTA) to identify most important areas relevant to working memory function. This method was first described as a tool for better assessment of hemispheric dominance for language [14]. The CTA approach is conceptually similar to the conjunction analysis but in this method only one factor is constructed for the "main effect" of task processing. This analysis helps us to report simi-

Table 2
Comparison of activations between the tasks; regardless of age

| Contrast | Region | Max Z stat | MNI coordinate (x, y, z) |
|---|---|---|---|
| Visual > Verbal | | | |
| | Lt Planum Temporale | 5.82 | (−62, −14, 8) |
| | Lt Middle frontal gyrus | 5.61 | (−33, 20, 32) |
| | Lt Middle temporal gyrus | 5.54 | (−54, −28, −10) |
| | Rt Frontal Pole | 5.19 | (24, 38, 32) |
| | Rt Superior frontal gyrus | 5.14 | (14, 24, 50) |
| | Lt Paracingulte gyrus | 5.10 | (−12, 46, 26) |
| Verbal > Visual | | | |
| | | N.S | |
| Visual > Auditory | Lt Cingulate gyrus, Post. division | 6.06 | (−6, −38, 0) |
| | Rt Lateral occipital cortex | 5.32 | (44, −72, 4) |
| | Lt Middle frontal gyrus | 5.14 | (−32, 16, 34) |
| | Lt Lingual gyrus | 5.12 | (−8, −60, 0) |
| | Lt Insular cortex | 4.98 | (−38, −4, 10) |
| Auditory > Visual | | | |
| | Rt Superior temporal gyrus | 6.11 | (55, −30, 9) |
| | Lt Heschl's gyrus | 4.98 | (−41, −16, 6) |
| | Rt Heschl's gyrus | 5.59 | (45, −22, 10) |
| Verbal > Auditory | | | |
| | Rt Lingual gyrus | 5.56 | (20, −58, 2) |
| | Lt Intracalcarine cortex | 5.54 | (−22, −70, 12) |
| | Rt Lateral occipital cortex | 5.38 | (36, −64, 10) |
| | Lt Lingual gyrus | 5.34 | (−16, −60, 4) |
| Auditory > Verbal | | | |
| | Lt Central opercular cortex | 6.21 | (−60, −10, 10) |
| | Lt Heschl's gyrus | 6.11 | (−40, −20, 8) |
| | Rt Thalamus | 5.11 | (22, −14, −6) |
| | Lt Postcentral gyrus | 5.87 | (−58, −8, 18) |
| | Rt Superior temporal gyrus | 5.85 | (60, −32, 8) |
| | Rt Heschl's gyrus | 5.83 | (54, −16, 4) |

N.S = Not Significant. Post = Posterior; Rt = Right; Lt = Left.

lar activations and convergence of activation among all working memory tasks. Higher-level analysis for CTA study (intra subject) was carried out using a fixed effect model, by forcing the random effect variances to zero. For the group analyses, activation surviving a corrected threshold of $p < 0.05$ was considered statistically significant.

Clusters of activation containing more than 10 voxels were localized by means of the Harvard–Oxford Cortical and Subcortical Structural Atlases (http://www.cma.mgh.harvard.edu/fsl_atlas.html, 15). Probability maps of the used atlases were first registered to MNI space. To document a meaningful comparison between the exploited tasks, we used a same threshold across all tasks.

## 3. Results

### 3.1. Behavioral results

Repeated measures ANOVA examined the effect of age group (between subject factor) and task type (3-level within subjects). There was a significant effect of task, $F(2,66) = 7.5$, $p = 0.01$, on accuracy of the responses. Subjects attained a mean score of 96.2% correct (SD 2.3, range 88–100%) for both visual and auditory tasks while their score for the verbal task was 81.4 (SD 4.5, range 69–100%). The main effect of age on accuracy did not produce a statistically significant result $F(1,33) = 0.038$, $p > 0.05$); a finding that shows a uniform and good task performance for both age groups. Moreover, the interaction of task and group was also not significant, $F(2,66) = 0.321$, $P > 0.05$.

Task-type exerted a significant effect on RT, $F(2,66) = 109.96$, $P < 0.0001$. Verbal task showed mean response time of 1226 ± 287 ms (mean ± standard deviation), which was longer relative to visual and verbal tasks (994 ± 183 ms and 872 ± 110 ms respectively). Our results showed that the main effect of group was not significant, $F(1,33) = 0.008$, $P > 0.05$. The average RT for the older subjects in all three tasks were 1023 ± 247 ms while this time for the young group was 998 ± 332 ms. The two-way interaction of task and group also was not significant, $F(2,66) = 0.016$, $P > 0.05$.

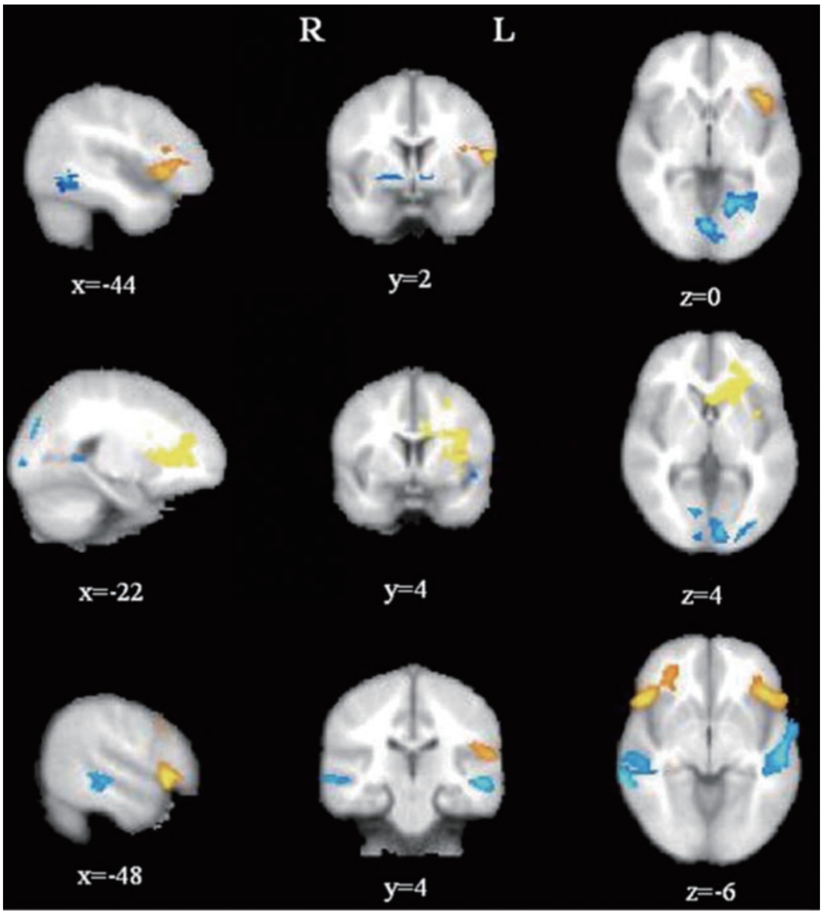

Fig. 3. Age-related activations visual (top), verbal (middle) and auditory (bottom) working memory tasks. Hot colors point to positive (older > younger) activations and cold colors negative activations (younger > older) for each contrast. Sagittal sections show left hemisphere. All maps are thresholded at a whole-brain corrected $P < 0.05$ using cluster-wise $P < 0.05$. For details see Table 3.

### *3.2. Within group analyses*

Widespread cortical activations were seen in all three tasks representing global operation of brain in working memory processing. In the visual working memory task, bilateral occipital activations in angular gyrus, lateral occipital cortex, and occipital pole were observed. Moreover, right superior and middle frontal gyri, as well as anterior division of cingulate gyrus was observed to be active in this task.

Verbal working memory task shared similar cortical activations in frontal and occipital lobes with the visual task but also revealed significant activations in right frontal pole, left thalamus and inferior frontal gyrus.

Our findings for the auditory working memory task elicited bilateral activation in superior temporal gyrus. A network of right frontal regions including frontal pole, superior frontal gyrus, middle frontal gyrus and precentral gyrus was observed to be active in the auditory task. This task also disclosed significant activations in left parietal operculum cortex and anterior division of cingulate gyrus. In contrast to the visual and verbal tasks, the auditory task requires participants to attend to auditory signals and hence this task lacks activations in visual cortices (Fig. 2 and Table 1).

Combined Task Analysis showed that frontal regions are shared activations across all three tasks.

### *3.3. Task comparisons and;ge-independent activations*

Results for each of the main task contrasts were obtained in between group analyses. Activation during the visual task compared to the verbal task, occurred mainly in frontal regions including left middle frontal gyrus, right frontal pole and right superior frontal gyrus.

Table 3
Age-related activations in each task

| Task | | Region | Max Z stat | MNI coordinate |
|---|---|---|---|---|
| Visual WM | | | | |
| | Young > old | Lt Precuneus cortex | 5.97 | (−12, −66, 18) |
| | | Rt Intracalcarine cortex | 5.86 | (8, −60, 18) |
| | Old>Young | Lt Inferior Frontal gyrus | 6.0 | (−44, 14, −2) |
| Verbal WM | | | | |
| | Young>old | Lt Lateral Occipital cortex | 6.2 | (−64, −36, 4) |
| | Old>Young | Lt Inferior Frontal gyrus | 4.64 | (−40, 18, 10) |
| Auditory WM | | | | |
| | Young>old | Rt Superior Temporal gyrus | 4.6 | (58, −20, −4) |
| | | Lt Superior Temporal gyrus | 5.9 | (−62, −24, −4) |
| | Old>Young | Lt Inferior Frontal gyrus | 5.1 | (−48, 20, 4) |
| | | Rt Inferior Frontal gyrus | 4.6 | (52, 20, 4) |
| | | Anterior Cingulated gyrus | 6.59 | (12, 44, 4) |

WM = working memory; Rt = Right; Lt = Left.

Moreover left middle temporal gyrus appeared to be more active in the visual task. There were no regions in which activation for the verbal task was greater than that in the visual task. This might be attributed to the same route of task presentation in both visual and verbal tasks Moreover, this finding may be due to a verbal strategy of the subjects for the visual task.

Compared to the auditory task, the visual task produced relative BOLD signal enhancement in lateral occipital cortex, lingual gyrus, insular cortex and left middle frontal gyrus. In Auditory-visual contrast, bilateral Heschl's gyri and right primary auditory cortex showed significant activations.

Occipital areas comprising lingual gyrus, intracalcarine cortex and lateral occipital cortex had greater activations for the verbal than for the auditory task whereas Heschl's gyrus, right superior temporal gyrus, right thalamus, and central opercular cortex activated more for the auditory than the verbal task. A summary of task comparisons can be found in Table 2.

### *3.4. Task-specific age effects*

Considering age as an additional covariate, we looked for positive and negative impacts of age on activations in each task. Our results showed that left inferior frontal gyrus is the only age-related activation across all three tasks and this area demonstrates stronger activation in the older group. An interesting task-specific age effect was that during visual and verbal tasks the visual cortices were less activated in older adults than in their younger counterparts (see Fig. 3). Other task-specific age effects involved age-related decreases in primary auditory cortex activity during the auditory WM task (Table 3).

## 4. Discussion

Our study revealed three key findings. First, the results show that regardless of the stimulus used to activate the working memory circuit, there are significant activations in prefrontal regions, although anatomic segregations are also apparent. Secondly, our results indicate that direct contrasting between the tasks typically result in regions that are related to processing of that specific stimulus type. Remarkably, this study directly compared a new and novel auditory stimulus with the two frequently studied stimuli; verbal and visual tasks, and showed that the activations in this task are comparable to those of visual and verbal stimuli. Finally, we found an age-related reduction in activity of visual and auditory cortices while an age-related increase in prefrontal cortex activity was also evident.

One of the main controversies in the literature with regard to functional imaging of working memory is whether there is an anatomical segregation on the basis of stimulus category [4]. The other aspect of the controversial issues in working memory studies is categorizing the organization of working memory by means of process type (i.e. maintenance or manipulation of the memory contents). To be able to compare different stimuli in this study, we utilized a similar design for all the stimuli to ensure eliminating confounding impacts of process type. Considering the dual pathway for processing of spatial and non-spatial (object) working memory, we used non-spatial designs in all of the three tasks. Our findings for the visual and verbal tasks are comparable to previous studies. Modality-specific activations, with occipito-temporal activation in visual working memory tasks have been formerly described [9]. In our study, we used Persian alphabet as the stimuli in a verbal Sternberg task. Persian, unlike Arabic and Hebrew, which are Semitic languages is an Indo-European language but different from English and other Latin-based languages in many aspects. Persian consists of 29 consonants and 6 vowels. Some of the main differences are that it is written from right to left, letters are some attached and some detached from each other, and diacritics are only used for beginner readers [16]. This study just used consonants in detached form. Our results for verbal working memory with Persian letters are the same as a recent study with Latin alphabet in which frontal, insular, cingulate and bilateral inferior parietal/supramarginal regions were shown to be active [17]. These similarities suggest that language processing and maintenance for Persian is identical to English rather than Arabic. This linguistic finding was highlighted in previous reports [16]. Recent studies proposed common neural substrate for language production and verbal working memory [18,19]. This premise could be confirmed by our study since we showed left inferior frontal gyrus activation in the verbal working memory task. Activations in this area could be mainly attributed to activation of Broca's area.

For the auditory fMRI tasks, scanner noise is one of the most challenging issues because the activation observed in these tasks could occur as a result of the background noise. To overcome this problem we took advantage of masking properties of white noise. Our result for the auditory working memory showed significant activations in the superior temporal gyrus and right frontal regions. These findings are comparable to those of Arnott's et al. where sound identification was associated with greater hemodynamic activity in the left rostral superior temporal gyrus [20]. That study revealed dominant activations in dorsolateral prefrontal and lateral superior parietal areas during manipulative stage of their trial, which is in accordance with the frontal activations in our study.

Modality-specific regions for working memory have been reported; but there is increasing data for commonalities of regions involved in different types of working memory. Most of previous studies mainly compared letters, words, figures, and pictures as the triggering stimulus [21,22]. Our visual-verbal contrast showed frontal regions and left middle temporal gyrus activations. This contrast lacks activations in visual cortex since in our design; both visual and verbal cues were visually presented. Or findings in visual-auditory contrast are consistent with similar prior studies and shows activation favoring areas involved in visual processing. Comparing aurally and visually presented information for verbal working memory, Kirschen and colleagues came up with the same results. That study showed that visual presentation evoked prominent activations in bilateral occipital (BA19) and left parietal (BA7/40) corteices [17]. These results are also consistent with our next contrast, verbal-auditory, that showed lingual gyrus and intracalcarine cortex activations. Obviously, and as previous studies confirmed, auditory-verbal and auditory-visual contrasts in our study showed more activation primarily in auditory cortices.

In general, the contrasts showed that most of the observed differences between the tasks could be attributed to feature processing rather than working memory execution. According to our Combined Task Analysis, frontal regions showed to be a ceaseless activation area for all working memory tasks. This finding is in accordance with Baddeley's model for working memory as well as previous functional imaging studies [23,24].

One enduring controversy in the cognitive literature is the nature of age effects on cerebral functions. There is much evidence that age effects can vary considerably across tasks [25]. In the present study, we used functional neuroimaging techniques to understand task-specific and task-independent effect of aging on working memory processes. Our results indicate that activations in frontal regions in all three exploited tasks are age-related. This finding is of great value for clinical interpretations, since most of the usage of working memory tasks are to detect early cognitive impairments in patients with dementia, a disease that more commonly affects the elderly. Previous functional imaging studies of age-associated changes in brain activation patterns reveal altered activity in working memory tasks as well as the recruitment of additional regions as a person gets older [26–28]. As it is evident in Table 3 and Fig. 3, each task represents a unique age-related network. Specifically, the figure indicates that frontal regions are the only areas that show greater activation in older subjects in all three tasks (hot colors in the figure). This finding is the intriguing difference between the current study and previous reports, since there is paucity of data in the literature to show age-related activations in non-visual tasks. Moreover, Fig. 3 also reveals hypofunctioning of visual and auditory cortices in older subjects (blue-cyan).

Weaker visual cortex activity in older adults across visual and verbal tasks is in accordance with the theory that age-related sensory processing plays a major role in cognitive aging [8]. This theory is supported by evidence of strong correlations between age-related sensory/perceptual decline in visual tasks [8,29]. This explanation is also applicable for age-related decline in superior temporal gyrus across auditory task, since this area is devoted to auditory stimuli processing. As sensory decline might be responsible for hypoactivation of sensory cortices in older subjects in our study, one limitation of the present work is absence of comparing sensory thresholds between the two groups. This peripheral hypofunctioning of the sensory systems might impose hyperfunctioning of prefrontal regions to compensate for the increased sensory threshold. It has been suggested that some forms of functional compensation in older adults may share a common mechanism across different stimuli. Given that prefrontal cortex is employed by a variety of cognitive tasks [30,31] it is not unexpected to find this area as a mutual age-related activation across all exploited tasks. To date there are no studies to address primary auditory cortex as a target for age-related weakening in auditory tasks. Hence our findings in case of activations in frontal regions in auditory working memory task may imply a silent compensation for auditory cortex. Nonetheless it is established that age-related increases in prefrontal cortex activity could compensate for age-related decreases in visual cortex activity [8,32].

In conclusion, our findings could be summarized as follows. First, consistent with our predictions, frontal regions exhibit task and age–independent activations in working memory processes. Secondly, prefrontal activation is not confined to visually presented clues and could be recruited in auditory tasks. Finally, regardless of the utilized task, a constant pattern in age effects on working memory functions could be detected. This pattern comprises an age-related reduction in visual or auditory activity (depending on stimulus type) along with an age-related increase in prefrontal cortex. This finding might indicate that age-related reductions in sensory processing may contribute to cognitive deficits, and compensatory mechanisms of prefrontal cortex may try to counteract the sensory decline.

## Acknowledgment

The authors thank Kate Brown and Marjan Zakeri form University of British Columbia for valuable comments in preparation of the revised manuscript.
